# Development and validation of a genomic instability-related lncRNA prognostic model for hepatocellular carcinoma

**DOI:** 10.3389/fgene.2022.1034979

**Published:** 2023-01-12

**Authors:** Ziyu Xun, Yanyu Wang, Junyu Long, Yiran Li, Xu Yang, Huishan Sun, Haitao Zhao

**Affiliations:** State Key Laboratory of Complex Severe and Rare Disease Department of Liver Surgery, Peking Union Medical College Hospital, Chinese Academy of Medical Sciences & Peking Union Medical College, Beijing, China

**Keywords:** hepatocellular carcinoma, lncRNAs, prognosis, genomic instability, immune environment

## Abstract

Genomic instability is a characteristic of tumors, and recent studies have shown that it is related to a poor prognosis of multiple cancers. Long non-coding RNAs (lncRNAs) have become a research hotspot in recent years, and many unknown biological functions are being explored. For example, some lncRNAs play a critical role in the initiation and progression of multiple cancer types by modulating genomic instability. However, the role of genomic instability-related lncRNAs in liver cancer remains unclear. Therefore, we screened genomic instability-related lncRNAs by combining somatic mutation data and RNA-Seq data in The Cancer Genome Atlas (TCGA) database. We established a genomic instability-related lncRNA model (GLncM) involving *ZFPM2-AS1* and *MIR210HG* to predict the hepatocellular carcinoma (HCC) prognosis and further explore the clinical significance of these lncRNAs, and the robustness of the model was validated in the verification set. Thereafter, we calculated the immune score for each patient and explored the relationship between genome instability and the immune microenvironment. The analysis indicated that this model was better than the immune microenvironment in predicting the prognosis of HCC patients, suggesting that the GLncM may be an effective indicator of HCC prognosis and providing a new direction and strategy for estimating the prognosis of HCC patients.

## Introduction

Primary liver cancer is the sixth most commonly diagnosed cancer worldwide, and its mortality rate has increased to third (Sung et al., 2021). Primary liver cancer principally includes two histological types: hepatocellular carcinoma (HCC) and intrahepatic cholangiocarcinoma (ICC). Globally, the dominant histological type of primary liver cancer in most countries is HCC, accounting for approximately 75%–85% of all cases of primary liver cancer. In most high-risk areas, including China and Africa, the key risk factors for HCC are chronic hepatitis B virus (HBV) infection and aflatoxin exposure (Sung et al., 2021). In addition to traditional radiotherapy and chemotherapy, targeted therapy and immunotherapy have recently emerged as new treatments for HCC(Kudo et al., 2018; Finn et al., 2020); although these treatments have rapidly progressed, the prognosis of most patients with HCC is still unfavorable due to the heterogeneity of HCC(Allemani et al., 2018). Thus, more sensitive biomarkers must be identified to estimate the prognosis of HCC.

Genomic instability is a prevalent characteristic of most cancers, from precancerous lesions to advanced tumors (Li et al., 2021; Chen et al., 2022). In HCC, HBV-DNA integrates into the host genome at the early stage of tumor amplification and induces genome instability (Levrero and Zucman-Rossi, 2016). Indeed, numerous studies have indicated that genetic instability is related to a poor prognosis of cancers. It has also been reported that the genetically unstable type of breast cancer has a higher risk of significantly shorter recurrence-free survival and metastasis-free survival than other types (Duijf et al., 2019). Consistent with previous reports, genomic instability is also associated with a poor prognosis in colorectal cancer, ovarian cancer and other tumors (Xu et al., 2021; Lakbir et al., 2022). However, studies have shown that extreme genetic instability is correlated with a better prognosis (Andor et al., 2017).

Long non-coding RNAs (lncRNAs) are a class of non-protein-coding RNA molecules whose transcripts are more than 200 nucleotides long (Ulitsky and Bartel, 2013). To date, genome-wide analyses have revealed more than 50,000 genes that transcribe lncRNAs, and their number is still increasing rapidly (Iyer et al., 2015). LncRNAs were initially considered RNAs without biological functions, but researchers have found that some lncRNAs play key roles in tumorigenesis and progression through epigenetic regulation, DNA damage and cell cycle regulation, microRNA regulation, and participation in signal transduction pathways (Hu et al., 2018; Liu et al., 2018; Wang R. et al., 2018; Wang Z. et al., 2018; Huang et al., 2019). For example, H19, HOTAIR and HULC have been shown to facilitate the invasion and metastasis of HCC(Ding et al., 2014; Li et al., 2014; Li D. et al., 2017; Lv et al., 2017; Wang et al., 2017).

In this article, we combined genetic instability with lncRNAs, classified patients in The Cancer Genome Atlas (TCGA)-LIHC database according to lncRNA expression levels and somatic mutations, and analyzed clinical characteristics to construct an HCC prognostic risk model.

## Results

### Classification of genomic instability-related lncRNAs in HCC patients

**FIGURE 1:**
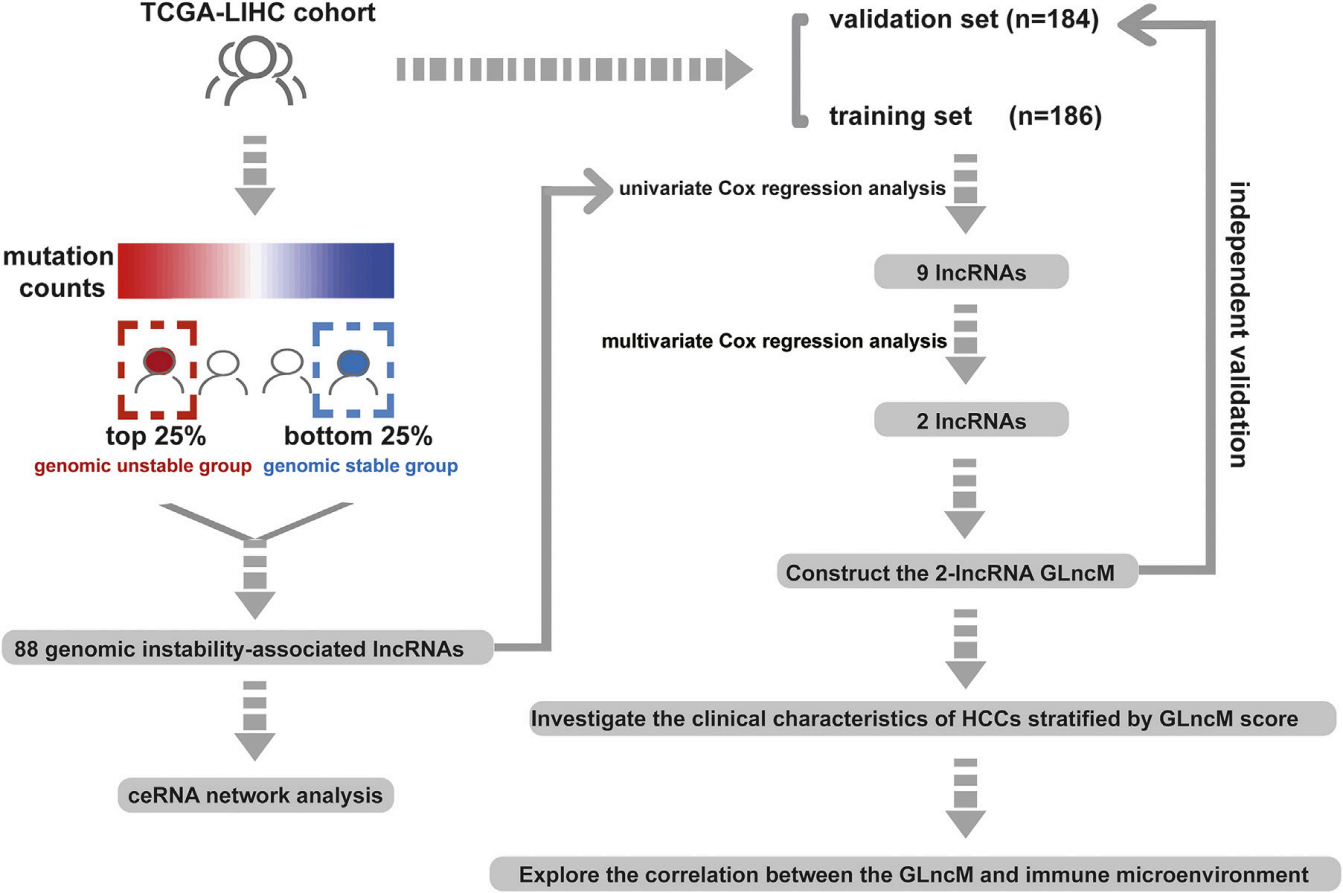
Workflow of this study.

**FIGURE 2:**
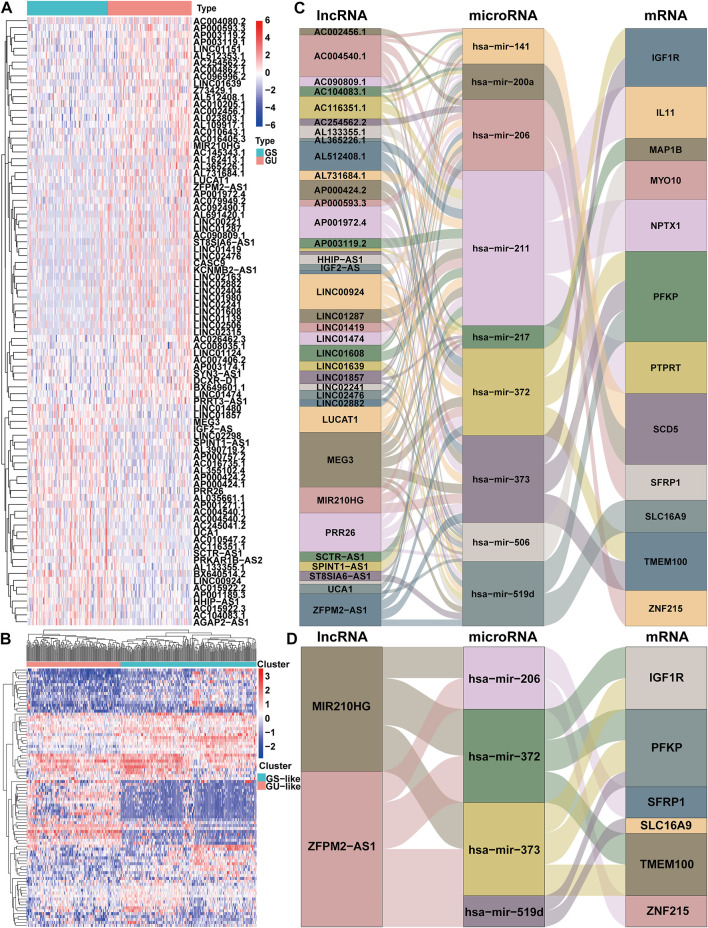
Identification and ceRNA network analysis of genomic instability-associated lncRNAs in HCC. **(A)** Differential expression of 88 genomic instability-associated lncRNAs between the genomic stable set and genomic unstable set **(B)** Unsupervised clustering analysis of 364 HCC samples according to the expression patterns of 88 selected lncRNAs correlated with genomic instability. Sankey diagram of the ceRNA network of all lncRNAs correlated with genomic instability **(C)** and Sankey diagram of the ceRNA network of *ZFPM2-AS1* and *MIR210HG* **(D)**. The connection degree of each gene represented by a rectangular box is proportional to the size of the rectangle. GS: genomic stable; GU: genomic unstable.

The workflow describing the study is shown in Figure 1. We first calculated the total number of mutations in 364 HCC samples and then ranked samples in ascending order to obtain genomic instability-related lncRNAs. The top 25% of the samples (*n* = 90) were selected as the genomic stable group, and the bottom 25% (*n* = 93) were selected as the genomic unstable group. The number of mutations in the genomic stable group ranged from 3 to 78, and the number of mutations in the genomic unstable group ranged from 154 to 2055. Then, we performed a differential expression analysis using the Wilcoxon rank-sum test. As shown in Figure 2A, 88 lncRNAs were differentially expressed between the genomic stable group and the genomic unstable group and were regarded as genomic instability-related lncRNAs. Fifty-six of these lncRNAs were upregulated and 32 lncRNAs were downregulated in the genomic unstable group. We then observed statistically significant differences in the mutation numbers between the genomic stable group and genomic unstable group. In addition, the genomic stable group had higher stromal scores, immune scores, and ESTIMATE scores and better outcomes than the genomic unstable group. Based on the 88 genomic instability-related lncRNAs identified above, 374 HCC samples were grouped into two clusters using an unsupervised hierarchical clustering analysis (Figure 2B). We then observed statistically significant differences in the mutation numbers between the two clusters (131.5 *versus* 102, *p* < 0.001, Wilcoxon rank-sum test, Supplementary Figure S1A). The group with a higher number of mutations was identified as the genomic unstable-like (GU-like) cluster (*n* = 153), and the group with a lower number of mutations was identified as the genomic stable-like (GS-like) cluster (*n* = 221).

We established a ceRNA network based on the lncRNA expression profiles, mRNA expression profiles and microRNA expression data and visualized the network using the ggalluvial R package (Version: 0.12.3) to better understand the function of 88 genomic instability-related lncRNAs (Figure 2C). Seventy genomic instability-related lncRNAs that interact with 86 differentially expressed microRNAs were searched from the miRcode database. Sixteen of these eighty-six differentially expressed microRNAs were obtained from the starBase database. Then, differentially expressed mRNAs were retrieved based on sixteen differentially expressed microRNAs in the miRDB, miRTarBase, and TargetScan databases. According to all three of the databases, twelve differentially expressed mRNAs interacted with nine of the sixteen differentially expressed microRNAs. Finally, 36 genomic instability-related lncRNAs, 9 differentially expressed microRNAs and twelve differentially expressed mRNAs were used to construct a ceRNA network (Figure 2C).

### Construction of a genomic instability-associated lncRNA prognostic model for HCC patients

We constructed a prognostic model based on these lncRNAs to further elucidate the prognostic value of genomic instability-associated lncRNAs. First, 370 HCC samples, excluding duplicate samples, were randomly split into a training set (*n* = 186) and verification set (*n* = 184) at a 1:1 ratio. The clinical features of the training set and verification set are displayed in Table 1. The distribution of each clinical feature between the training set and verification set was balanced. Next, a univariate Cox regression analysis of the training set was conducted to obtain 9 lncRNAs associated with the prognosis (*p* < 0.05; Table 2). Then, a multivariate Cox regression analysis was performed on these 9 lncRNAs to obtain two lncRNAs with independent prognostic value (*ZFPM2-AS1* and *MIR210HG*, *p* < 0.05, Table 2). Finally, a genomic instability-related lncRNA model (GLncM) was constructed using the expression levels and coefficients from the multivariate Cox regression analysis of *ZFPM2-AS1* and *MIR210HG* to evaluate the prognosis of HCC patients. The formula was as follows: GLncM score = 0.119315765508808 × expression level of *ZFPM2-AS1*+0.142471464922762 × expression level of *MIR210HG*. In the formula, the coefficients of *ZFPM2-AS1* and *MIR210HG* were both positive, indicating that these two genes were risk factors and that high expression of these genes was correlated with a poorer prognosis than low expression. Liu et al. and Wang et al. previously confirmed that high expression of the two lncRNAs was associated with the proliferation, migration and invasion of HCC cell lines. According to the previous ceRNA network, microRNAs that interact with *ZFPM2-AS1* and *MIR210HG* play a pivotal role in oncogenesis and tumor development (Figure 2D). For instance, Ren et al. previously reported that hsa-mir-206 inhibits tumor growth and metastasis by inhibiting the translation of the SFRP1 protein (Ren et al., 2014). The two lncRNAs in the GLncM may competitively bind to hsa-miR-206, thereby disrupting the binding of the microRNA and SFRP1 and leading to oncogenesis and tumor progression.

**TABLE 1:** Clinical features of the training cohort, testing cohort and whole TCGA-LIHC cohort.

Covariates		TCGA set (*n* = 370)	Training set (*n* = 186)	Testing set (*n* = 184)	*p*-value
Sex	Female	121 (32.7%)	66 (35.48%)	55 (29.89%)	0.3003
	Male	249 (67.3%)	120 (64.52%)	129 (70.11%)	
Age	≤61	192 (51.89%)	94 (50.54%)	98 (53.26%)	0.6744
	>61	178 (48.11%)	92 (49.46%)	86 (46.74%)	
Family history	No	90 (24.32%)	49 (26.34%)	41 (22.28%)	0.43
	Yes	280 (75.68%)	137 (73.66%)	143 (77.72%)	
Stage	Stage I-II	256 (69.19%)	132 (70.97%)	124 (67.39%)	0.0975
	Stage III-IV	90 (24.32%)	45 (24.19%)	45 (24.46%)	
	Unknown	24 (6.49%)	9 (4.84%)	15 (8.15%)	
Child-pugh classification	A	216 (58.38%)	109 (58.6%)	107 (58.15%)	0.0975
	B-C	22 (5.95%)	11 (5.91%)	11 (5.98%)	
	Unknow	132 (35.68%)	66 (35.48%)	66 (35.87%)	
Grade	G1-G2	232 (62.7%)	114 (61.29%)	118 (64.13%)	0.125
	G3-G4	133 (35.95%)	69 (37.1%)	64 (34.78%)	
	Unknow	5 (1.35%)	3 (1.61%)	2 (1.09%)	
AFP	<400	213 (57.57%)	109 (58.6%)	104 (56.52%)	0.9823
	>400	64 (17.3%)	32 (17.2%)	32 (17.39%)	
	Unknow	93 (25.14%)	45 (24.19%)	48 (26.09%)	

**TABLE 2:** Univariate and multivariate Cox regression analysis of the 88 genomic instability-associated lncRNAs correlated with OS in the training cohort.

Gene symbol	Univariate cox regression analysis result					Multivariate cox regression analysis result				
	Coefficient	HR	HR.95L	HR.95H	*p*-value	Coefficient	HR	HR.95L	HR.95H	*p*-value
LINC00221	0.138	1.148	1.028	1.282	0.015					
ZFPM2-AS1	0.128	1.136	1.083	1.192	<0.001	0.119	1.127	1.068	1.189	0.000
AC145343.1	0.341	1.407	1.150	1.720	0.001	0.183	1.201	0.953	1.515	0.121
KCNMB2-AS1	0.192	1.212	1.041	1.411	0.013					
PRRT3-AS1	0.093	1.098	1.012	1.191	0.025					
ST8SIA6-AS1	0.087	1.091	1.000	1.190	0.049					
LUCAT1	0.276	1.318	1.129	1.540	<0.001					
MIR210HG	0.197	1.218	1.109	1.338	<0.001	0.142	1.153	1.032	1.289	0.012
CASC9	0.070	1.072	1.029	1.118	0.001					

HR,hazard ratio; OS, overall survival.

**FIGURE 3:**
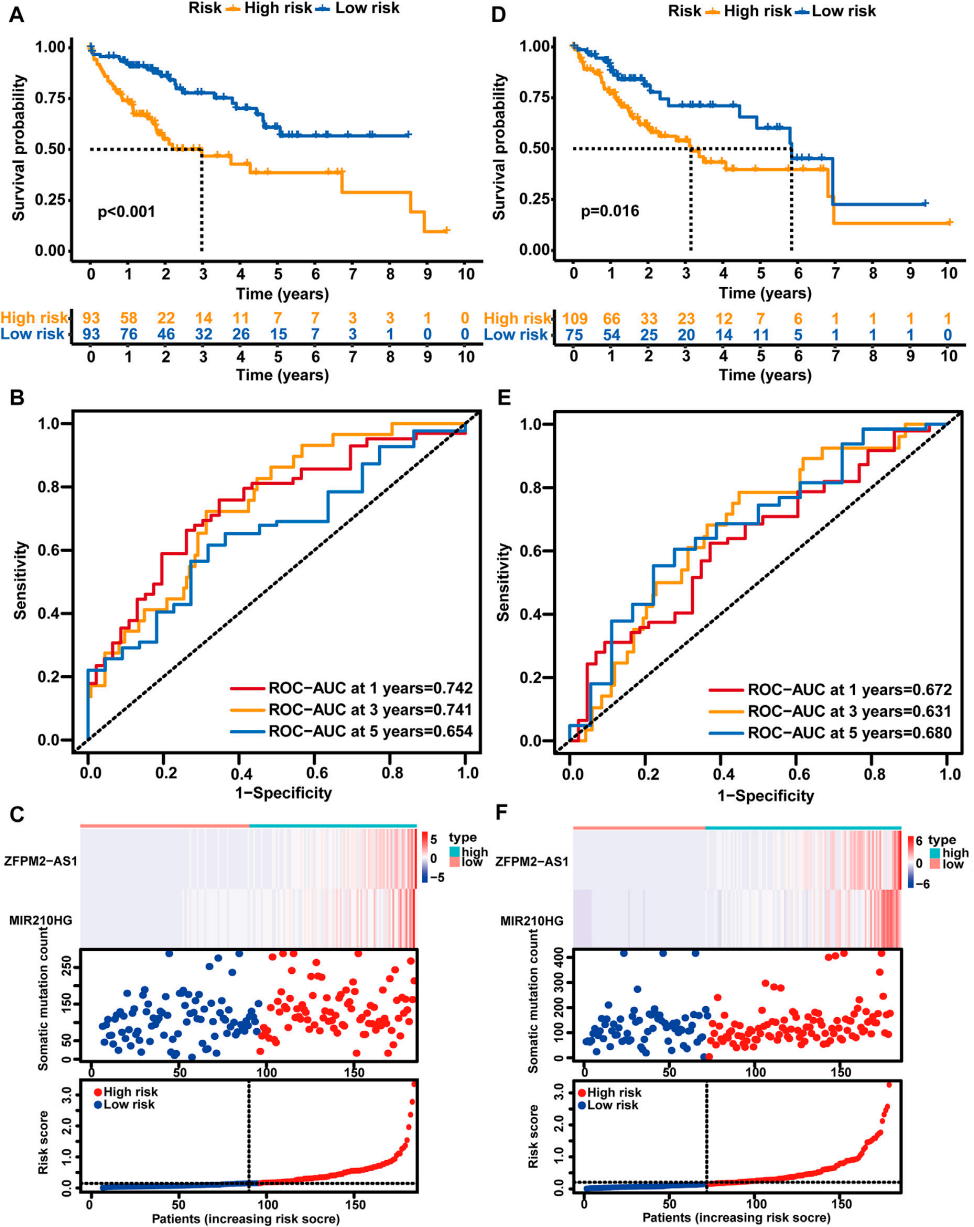
Analysis of the prognostic performance of the GLncM. Kaplan‒Meier survival analysis of the OS of the high-risk and low-risk groups predicted by the GLncM in the training group **(A)** and verification group **(D)**. The log-rank test was applied for the statistical analysis. **(B)** and **(E)** Time-dependent ROC curves for the analysis of the performance of the GLncM at 1, 3, and 5 years in the training group and verification group, respectively. **(C)** and **(F)** The lncRNA expression profiles, the risk score and the landscape of mutation counts in the training group and verification group. GLncM: genomic instability-related lncRNA model; OS: overall survival; ROC: receiver operating characteristic.

Afterward, the risk score of each sample was calculated based on the GLncM, and the median value of the risk score (0.1484198) was regarded as the cutoff value to classify the samples into a high-risk group and a low-risk group. As presented in Figure 3A, the low-risk patients experienced a longer overall survival (OS) than their high-risk counterparts (median OS NA vs. 2.98 years, *p* < 0.001), and the hazard ratio (HR) of the OS of low-versus high-risk group was 0.394 (95% CI: 0.223-0.696). The area under the receiver operating characteristic (ROC) curve (AUC) of the GLncM for OS was 0.742 (95% CI: 0.652-0.833) at 1 year, 0.741 (95% CI: 0.639-0.844) at 3 years and 0.654 (95% CI: 0.52-0.787) at 5 years (Figure 3B). In addition, we ranked the patients according to the risk score in ascending order and displayed the expression patterns of *ZFPM2-AS1* and *MIR210HG* and the landscape of mutation counts and risk scores in the training set (Figure 3C).

### Validation of the GLncM in HCC patients

Using the same GLncM formula and cutoff value obtained from the training set, we classified patients in the verification set into high- and low-risk groups. Similar to the result for the training set, the high-risk group experienced a noticeably shorter OS than the low-risk group (median OS 3.15 vs. 5.84 years, *p* = 0.016, Figure 3D). The AUC of the GLncM for OS was 0.672 (95% CI: 0.569-0.776) at 1 year, 0.631 (95% CI: 0.515-0.748) at 3 years and 0.680 (95% CI: 0.532-0.829) at 5 years (Figure 3E), and the HR of the OS of the low-versus high-risk group was 0.456 (95% CI: 0.256-0.815). Moreover, we ranked the patients according to the risk score in ascending order and described the *ZFPM2-AS1* and *MIR210HG* expression patterns and the landscape of mutation counts and risk scores in the testing set (Figure 3F).

Additionally, we stratified all patients in TCGA-LIHC cohort into high- and low-risk patient groups based on the GLncM and the same cutoff value acquired from the training set. Similar to the results described above, patients in the high-risk group experienced a shorter OS than their low-risk counterparts (median OS 3.11 vs. 6.94 years, *p* < 0.001, Supplementary Figure S1B). The AUC of the GLncM for OS was 0.709 (95% CI: 0.641–0.778) at 1 year, 0.687 (95% CI: 0.610–0.765) at 3 years and 0.666 (95% CI: 0.568–0.765) at 5 years (Supplementary Figure S1C), and patients in the low-risk group had a lower risk than those in the high-risk group (HR: 0.413, 95% CI: 0.276–0.618). Based on the ascending order of risk scores of patients, we displayed the *ZFPM2-AS1* and *MIR210HG* expression patterns, mutation counts and risk scores for the whole TCGA-LIHC dataset (Supplementary Figure S1D).

### Independence of the GLncM from other clinical factors

**FIGURE 4:**
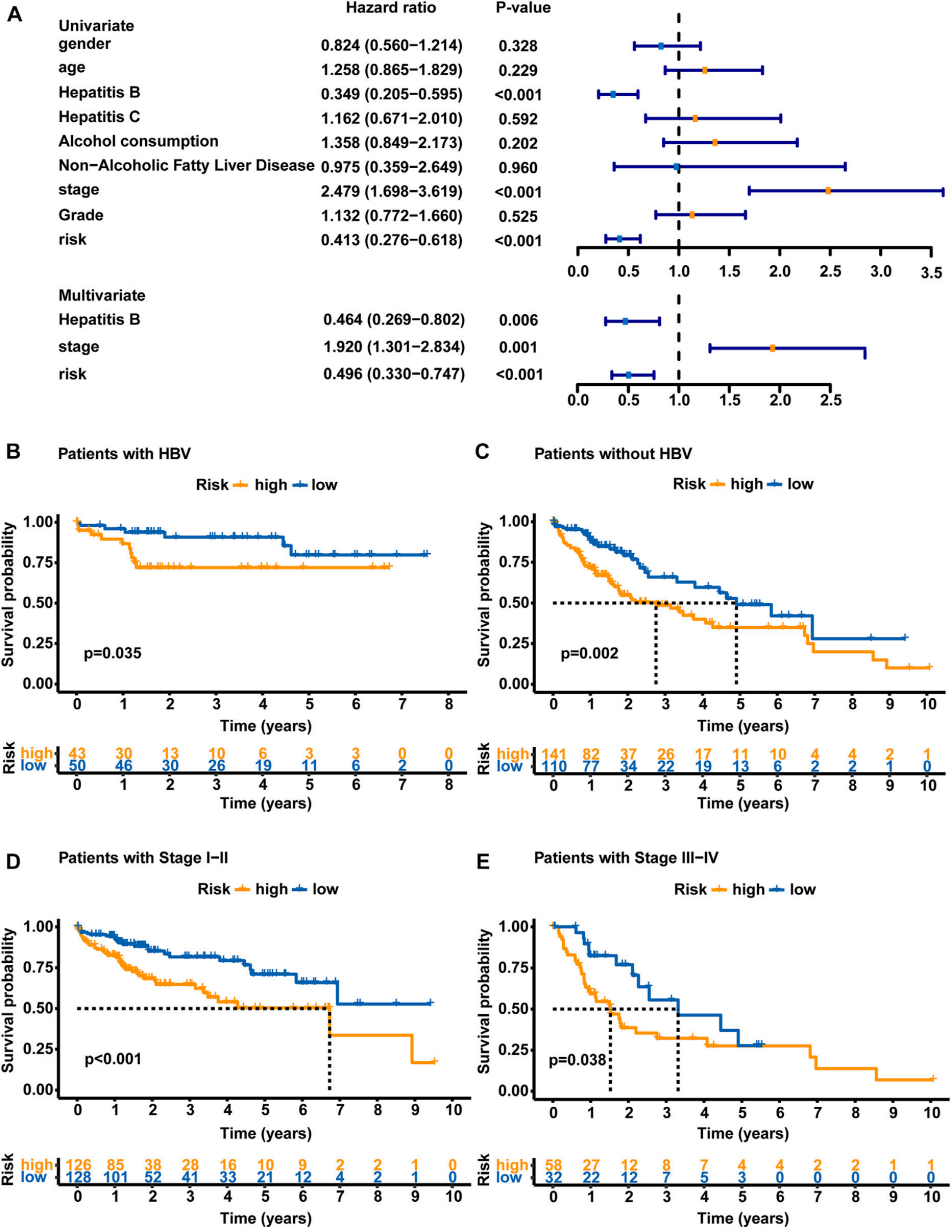
Analyses stratified according to the HBV status and tumor stage. **(A)** Univariate and multivariate Cox regression analyses of the GLncM and clinical characteristics of the whole TCGA-LIHC cohort. Only factors with *p* values less than 0.05 in the univariate analysis were included in the multivariate analysis. OS time illustrated by Kaplan-Meier curves of the high-risk and low-risk subgroups of HCC patients with HBV **(B)** and without HBV **(C)**. OS time illustrated by Kaplan-Meier curve in high- and low-risk subgroups of stage I-II patients **(D)** and stage III-IV patients **(E)**. The log-rank test was applied for the statistical analysis. HBV, hepatitis B virus; OS, overall survival.

Clinical features, including sex, age, HBV infection status, hepatitis C virus (HCV) infection status, non-alcoholic fatty liver disease, alcohol consumption, pathologic stage and histologic grade, were subjected to a univariate Cox regression analysis and multivariate Cox regression analyses with the established GLncM to assess whether the GLncM was independent of common clinical features. As shown in Figure 4A and Supplementary Table S1, the results of univariate and multivariate Cox regression analyses showed that GLncM was an independent factor for estimating the prognosis of HCC. In addition, the *p* values of the other two clinical features, HBV infection status and pathologic stage, were statistically significant in both the univariate and multivariate Cox analyses. Thus, we applied a stratification analysis to verify whether the prognostic value of the GLncM was independent of the HBV infection status and pathologic stage. TCGA-LIHC cohort was classified into the HBV-affected group (*n* = 251) and the non-HBV-affected group (n = 93). According to the GLncM and cutoff value identified previously, patients in each HBV infection status group were divided into high- and low-risk subgroups. As described in Figure 4B, a considerable difference in OS was observed between the high- and low-risk subgroups of the HBV-affected group (median OS 2.75 vs. 4.91 years, *p* = 0.035), and similar results were observed in the non-HBV-affected group (median OS NA vs. NA years, *p* = 0.002, Figure 4C). Next, patients in TCGA-LIHC dataset were divided according to pathologic stage; patients with stage I or II tumors were grouped into stage I - II (*n* = 254), and those with stage III or IV tumors were grouped into stage III - IV (*n* = 90). The GLncM with the cutoff value acquired previously divided the patients in the stage I–II group into a high-risk subgroup (*n* = 126) and a low-risk subgroup (*n* = 128), and the high-risk patients experienced a shorter OS than the low-risk patients (median OS 6.73 vs. NA years, *p* < 0.001, Figure 4D). Similarly, the GLncM was also applied to divide patients in the stage III - IV group into a high-risk subgroup (*n* = 58) and a low-risk subgroup (*n* = 32), and a significant difference in OS was observed between the two groups (median OS 1.52 vs. 3.52 years, *p* = 0.021, Figure 4E). Based on these results, the GLncM was an independent prognostic factor for HCC.

### Comparison of the GLncM with published lncRNA models in terms of prognostic prediction performance

We further assessed the predictive performance of GLncM by comparing it with two existing lncRNA-related models using the whole TCGA-LIHC dataset: the 5 lncRNA-based model from the study the study by Hou (HouLncM) (Hou et al., 2018) and the 5 lncRNA-based model from the study by Sun (SunLncM) (Sun et al., 2019).

**FIGURE 5:**
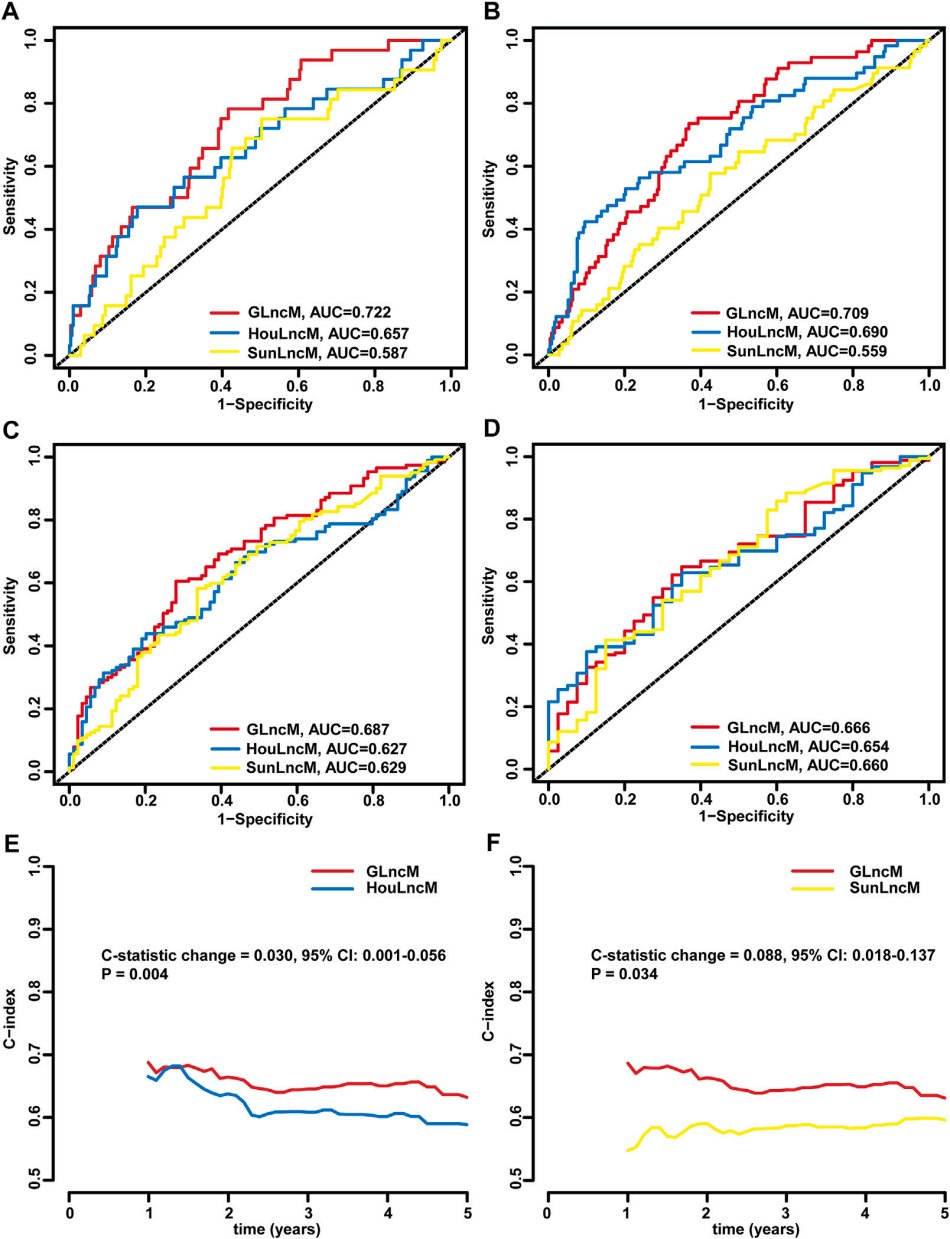
Comparison of the performance of GLncM with other models. ROC analysis at 0.5 years **(A)**, 1 year **(B)**, 3 years **(C)** and 5 years **(D)** of OS for GLncM, HouLncM and SunLncM. The C-statistic was applied to assess prognostic performance for predicting the OS of HCC patients. The change C-statistic change was calculated for GLncM and HouLncM **(E)** and GLncM and SunLncM **(F)** in TCGA-LIHC cohort. GLncM: genomic instability-related lncRNA model; HCC: hepatocellular carcinoma; HouLncM: lncRNA-based model from the study by Hou; ROC: receiver operating characteristic; SunLncM: lncRNA-based model from the study by Sun.

As shown in Figure 5A, the AUC of GLncM for OS was 0.722 at 0.5 years, which was significantly higher than that of HouLncM (AUC = 0.657) and SunLncM (AUC = 0.587). As shown in Figure 5B, the AUC of GLncM for OS was 0.709 at 1 year, which was significantly higher than that of HouLncM (AUC = 0.690) and SunLncM (AUC = 0.559). The AUC of OS for GLncM was 0.687 at 3 years, which was significantly higher than that of HouLncM (AUC = 0.627) and SunLncM (AUC = 0.629) (Figure 5C). In addition, as shown in Figure 5D, GLncM exhibited higher AUC values (AUC = 0.666) than the other two models (AUC of HouLncM = 0.654, AUC of SunLncM = 0.660) in the whole TCGA-LIHC dataset at 5 years. Moreover, we calculated the C-statistic for GLncM, HouLncM and SunLncM using the CsChange R package, and GLncM improved the C-statistic by 0.030 (95% CI: 0.001–0.056, *p* = 0.004) compared to HouLncM (Figure 5E) and increased the C-statistic by 0.088 (95% CI: 0.018–0.137, *p* = 0.034) compared to SunLncM (Figure 5F).

### The correlation between the immune scores and risk score

Since genomic instability has been reported to play a critical role by creating a precancerous environment (e.g., activation of oncogenic pathways, suppression of oxidative stress responses, and inhibition of immune function) (Rao et al., 2017), we used an immune scoring algorithm similar to that reported by Zhang et al. (2020) to investigate whether the immune environment differed between high-risk patients and low-risk patients. After the immune score of each HCC sample was calculated, the whole TCGA-LIHC cohort was split into a high immune score group and a low immune score group based on the median immune score (0.183). As shown in Supplementary Figure S2A, the OS of the high immune score group was significantly longer than that of the low immune score group (5.84 vs. 3.76 years, *p* < 0.001). Then, we analyzed the distribution of immune scores among the different risk groups. Patients in the high-risk group showed a significantly lower immune score than patients in the low-risk group (median score: 0.114 vs. 0.661, *p* = 0.0017, Supplementary Figure S2B). Moreover, using Spearman’s correlation analysis, we showed that the immune score was negatively correlated with the risk score (Spearman’s correlation coefficient: R = −0.25, *p* < 0.001, Supplementary Figure S2C).

**FIGURE 6:**
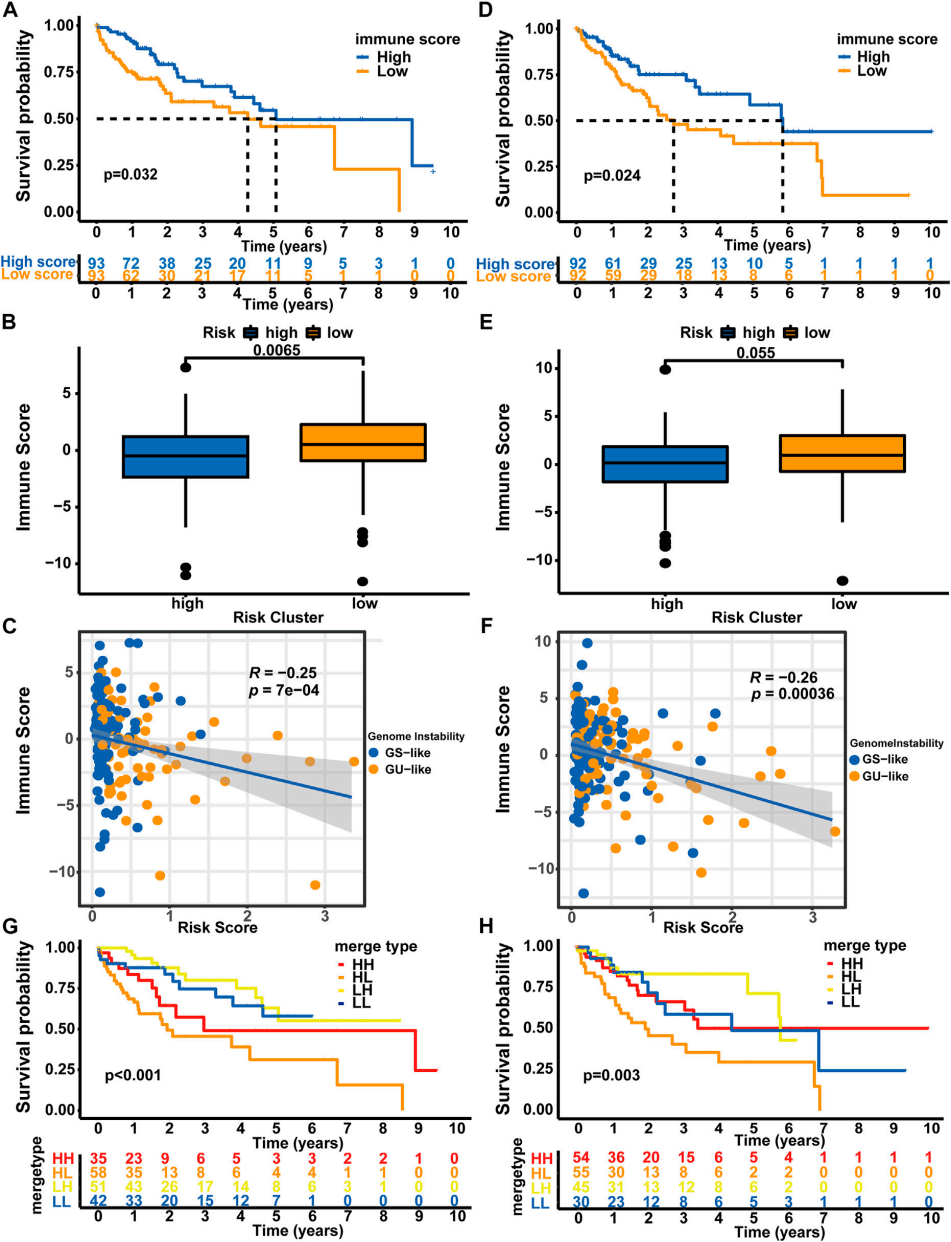
Combined analysis of GLncM and the immune microenvironment. Kaplan‒Meier curves for the high and low immune score groups in the training set **(A)** and verification set **(D)**. Difference in the immune scores between the high- and low-risk subgroups in the training set **(B)** and verification set **(E)**. Scatter plots showing the negative correlation between immune scores and risk scores in the training set **(C)** and testing set **(F)**. Kaplan‒Meier curves for patients in the training set **(G)** and verification set **(H)** stratified by both the immune score and risk score. The log-rank test and Spearman’s correlation analysis were applied for statistical analyses. GS-like: genomic stable-like; GU-like: genomic unstable-like; HH: high risk score and high immune score; HL: high risk score and low immune score; LH: low risk score and high immune score; LL: low risk score and low immune score.

Afterward, we analyzed the samples from the training set and verification set. Consistent with the results from the whole TCGA-LIHC dataset, patients in the high immune score group from the training set experienced a longer OS than those in the low immune score group (median OS: 5.07 vs. 4.27 years, *p* = 0.032, Figure 6A). Patients in the high-risk group showed a significantly lower immune score than patients in the low-risk group (median score: 0.497 vs. 0.520, *p* = 0.0065, Figure 6B), and correlation analyses confirmed that the immune score was negatively associated with the risk score (Spearman’s correlation coefficient: R = -0.25, P = 7e-04, Figure 6C). In the testing set, the OS of the low immune score group was significantly shorter than that of the high immune score group (median OS: 2.75 vs. 5.84 years, *p* = 0.024, Figure 6D). Patients in the high-risk group showed a significantly lower immune score than patients in the low-risk group (median score: 0.181 vs. 0.973, *p* = 0.055, Figure 6E), and correlation analyses confirmed that the immune score was negatively correlated with the risk score (Spearman’s coefficient: R = -0.26, *p* = 0.00036, Figure 6F). We then performed a combined survival analysis according to immune scores and risk scores in the training set, verification set and whole TCGA-LIHC dataset. Figures 6G,H and Supplementary Figure S2D show that patients with high immune scores in the low-risk group experienced a longer OS than other patients. Therefore, the GLncM score has greater prognostic significance than the status of the immune environment.

## Discussion

Currently, the prognosis of patients is mainly determined based on clinical characteristics and biochemical indicators, such as the tumor size, cirrhosis, tumor number, microvascular infiltration and Child‒Pugh score (Bertino et al., 2011; Chan et al., 2018; Galle et al., 2019; Huang and Gao, 2020). With the development of emerging detection technologies, researchers have begun to conduct in-depth research on tumors at the molecular level. Specific gene mutations, methylation status, microRNAs, *etc.*, have also been investigated to assess their potential for predicting the prognosis of the disease (Xu et al., 2017; Long et al., 2018; Long et al., 2019). However, the complex relationship between heredity, etiology and environmental risk factors results in genotypic and phenotypic heterogeneity in HCC and has increased the difficulty of determining the prognostic subtypes of HCC(Herath et al., 2006; Feo et al., 2009). Therefore, a more flexible and accurate prognostic model must be developed. With the emergence of high-throughput sequencing technology and microarray analyses, recent studies on lncRNAs have found that they play a role in different mechanisms regulating gene expression. Based on accumulating evidence, lncRNAs are involved in regulating proliferation, apoptosis, invasion and angiogenesis during the occurrence and development of liver cancer (Li et al., 2016; Wang et al., 2016; Zhang et al., 2016). At present, few studies on genomic instability and lncRNAs in liver cancer have been conducted, and the exact biological function and molecular mechanism of related lncRNAs are still unknown. Therefore, further exploration and verification are needed to clarify the complex mechanism.

We performed a combined analysis of gene expression data and somatic mutation data and obtained 88 genomic instability-related lncRNAs. Based on a ceRNA network analysis, we observed that lncRNAs may interrupt the regulatory effect of microRNAs on mRNAs to promote HCC oncogenesis and progression. Several lncRNAs among these genomic instability-related lncRNAs inhibit metastasis progression. For example, hsa-miR-204 downregulates IL-11 to inhibit the bone metastasis of breast cancer (Pollari et al., 2012).

We further assessed whether lncRNAs correlated with genomic instability predicted the clinical outcomes of HCC patients and acquired a lncRNA model consisting of two lncRNAs correlated with genomic instability (*ZFPM2-AS1* and *MIR210HG*). *ZFPM2-AS1* and *MIR210HG* have previously been reported to be involved in cell proliferation and migration (Li J. et al., 2017; Kang et al., 2019; Wang Y. et al., 2019; He et al., 2020; Liu et al., 2020; Wang et al., 2020), indicating that the model composed of these two lncRNAs is not only an indicator of genomic instability, but also can predict the prognosis in patients with HCC. Based on the results of the ceRNA network analysis, the two lncRNAs included in the GLncM may promote HCC oncogenesis and progression by competing with microRNAs for the binding of mRNAs. For example, the correlation between hsa-miR-372 and TMEM100 plays an important role in the oncogenesis and progression of several types of cancer, including bladder cancer, colorectal cancer, and gastric cancer (Fan and Liu, 2018; Pan et al., 2019; Pang et al., 2019; Wang J. et al., 2019). *ZFPM2-AS1* and *MIR210HG* may disrupt the interaction between hsa-miR-372 and TMEM100 to promote the development of HCC, but the function of hsa-miR-372 and TMEM100 in HCC has not been reported, and thus further experiments are needed to confirm this hypothesis. Furthermore, *ZFPM2-AS1* and *MIR210HG* may also promote the development of HCC by interrupting the interaction between hsa-miR-372 and PFKP, since PFKP has previously been reported to play an important role in cell proliferation (Moon et al., 2011).

Next, we evaluated the synergistic effect of the risk score and immune score on the prognostic stratification of HCC. The results indicated that genomic instability affected the immune microenvironment, but the immune status did not interfere with predictions obtained with the genomic instability-based model.

Currently, reliable prognostic biomarkers for HCC are still lacking. We developed a lncRNA model associated with genomic instability as an independent prognostic marker to stratify HCC patients at risk, which will help clinicians analyze the prognosis of patients using more dimensions and provide new insights into the treatment of patients. Because all samples in this study were collected from databases, future GLncM validation will be conducted in a prospective multicenter cohort study. Our study still has some limitations. Since we did not identify a dataset containing both lncRNAs in the Gene Expression Omnibus (GEO) database, we did not use the external dataset to verify the model. More prospective datasets are needed for the validation and assessment of the performance of the model to ensure its robustness and reproducibility.

## Materials and methods

### Data collection

Data were acquired from TCGA database; these data included somatic mutation data for 364 primary HCC samples, microRNA expression profiles for 375 primary HCC samples, and gene fragments per kilobase of transcript per million reads mapped (FPKM) expression profiles and clinical characteristics for 374 primary HCC samples. In the modeling process, only 370 samples with both mRNA expression data and clinical data were used for analysis. We annotated the gene symbols and gene types according to the Homo_sapiens.GRCh38.102. chr.gtf file.

### Classification of genomic instability-related lncRNAs

We used a method similar to that described by Bao et al. to determine the lncRNAs associated with genomic instability (Bao et al., 2020). First, the total number of mutations in each sample was calculated based on somatic mutation data. Then, the samples were sorted in ascending order according to the total number of mutations in each sample. Then, the first 25% of the samples were selected as the genomic stable group, and the last 25% were selected as the genomic unstable group. Then, the Wilcoxon rank-sum test was applied to compare the expression of each lncRNA between the two groups. Finally, genomic instability-related lncRNAs were obtained (false discovery rate (FDR) < 0.05, |log2fold change (FC)| > 1).

### Construction of the competing endogenous RNA (ceRNA) network

LncRNA expression data, mRNA expression data and microRNA expression data were used to construct the ceRNA network, and the network was visualized using the ggalluvial R package (Version: 0.12.3) to better illustrate the functions of lncRNAs associated with genomic instability.

The construction of the ceRNA network mainly included two steps. 1) The differentially expressed mRNAs and microRNAs between the genomic stable group and genomic unstable group were obtained using the Wilcoxon rank-sum test and edgeR package (Version: 3.24.3) (Robinson et al., 2010), respectively (FDR<0.05, |logFC|>1). 2) The miRcode database (Version: 11) (Jeggari et al., 2012) was applied to determine the interaction between genomic instability-associated lncRNAs and differentially expressed microRNAs. Interactions between differentially expressed microRNAs and differentially expressed mRNAs were determined using the miRDB (Version: 5.0), miRTarBase (Version: 6.1), and TargetScan (Version: 7.2) databases (Agarwal et al., 2015; Wong and Wang, 2015; Chou et al., 2018).

### Construction and validation of a genomic instability-related lncRNA prognostic risk model

First, HCC samples were randomly split into a training set and verification set. Then, in the training set, the prognostic value of genomic instability-related lncRNAs for OS was estimated by performing a univariate Cox regression analysis. The lncRNAs with *p* < 0.05 were screened in a subsequent multivariate Cox regression analysis. The lncRNAs with *p* < 0.05 in the multivariate Cox regression analysis were regarded as the final lncRNAs for model construction. Genomic instability-related lncRNAs were obtained by performing univariate and multivariate Cox regression analyses using the survival R package (Version: 3.1–12). The predictive power of the prognostic risk model was evaluated using the log-rank test and Kaplan‒Meier survival analysis.

### Immune-related analysis

CIBERSORT(Newman et al., 2015) was used to calculate the infiltration levels of immune cells in each sample. Then, we used a method similar to that described by Zhang et al. (2020) to calculate the immune score for each sample. First, the samples were classified into different immune subtypes based on the CIBERSORT results. Then, differentially expressed genes in the three immune subtypes were obtained using the Wilcoxon rank-sum test. Afterward, the samples were classified into different gene clusters based on these genes. Genes that were positively correlated with gene clusters were regarded as gene signature A; otherwise, they were regarded as gene signature B. The feature genes were identified using the Boruta R package (Version: 7.0.0) (Kursa and Rudnicki, 2010). Principal component analysis (PCA) was conducted for gene signature A and gene signature B of feature genes. The immune score of each sample was calculated by subtracting the score of the first principal component of gene signature A from the score of the first principal component of gene signature B.

## Data Availability

Publicly available datasets were analyzed in this study. This data can be found here: https://www.cancer.gov/about-nci/organization/ccg/research/structural-genomics/tcga.
