# Supplementary material for: Development and validation of a genomic instability-related lncRNA prognostic model for hepatocellular carcinoma
Source: Front Genet. 2023 Jan 12;13:1034979. doi: 10.3389/fgene.2022.1034979 (PMC9877230; doi:10.3389/fgene.2022.1034979)
Supplement: Supplementary file 1 [file Table1.DOCX]

| **Table S1.** Univariate and multivariate Cox regression analysis of the GLncM and other clinical characteristics correlated with OS in the two groups. | | | | | | | | | |
| --- | --- | --- | --- | --- | --- | --- | --- | --- | --- |
|  | Univariate Cox regression analysis result | | | |  | Multivariate Cox regression analysis result | | | |
|  | HR | HR.95L | HR.95H | P-value |  | HR | HR.95L | HR.95H | P-value |
| Training set (n=186) |  |  |  |  |  |  |  |  |  |
| Sex | 0.743 | 0.428 | 1.290 | 0.291 |  |  |  |  |  |
| Age | 1.756 | 1.008 | 3.059 | 0.047 |  | 1.093 | 0.596 | 2.004 | 0.773 |
| Hepatitis B | 0.327 | 0.147 | 0.730 | 0.006 |  | 0.480 | 0.206 | 1.120 | 0.090 |
| Hepatitis C | 0.857 | 0.304 | 2.413 | 0.770 |  |  |  |  |  |
| Alcohol consumption | 1.128 | 0.507 | 2.507 | 0.768 |  |  |  |  |  |
| Non-Alcoholic Fatty Liver Disease | 1.122 | 0.404 | 3.117 | 0.825 |  |  |  |  |  |
| Stage | 2.304 | 1.314 | 4.042 | 0.004 |  | 1.966 | 1.114 | 3.470 | 0.020 |
| Grade | 0.937 | 0.539 | 1.631 | 0.819 |  |  |  |  |  |
| GLncM | 0.394 | 0.223 | 0.696 | 0.001 |  | 0.483 | 0.262 | 0.891 | 0.020 |
| Testing set (n=184) |  |  |  |  |  |  |  |  |  |
| Sex | 0.912 | 0.521 | 1.598 | 0.749 |  |  |  |  |  |
| Age | 0.924 | 0.546 | 1.564 | 0.769 |  |  |  |  |  |
| Hepatitis B | 0.374 | 0.182 | 0.767 | 0.007 |  | 0.466 | 0.222 | 0.977 | 0.043 |
| Hepatitis C | 1.317 | 0.680 | 2.553 | 0.414 |  |  |  |  |  |
| Alcohol consumption | 1.377 | 0.755 | 2.509 | 0.297 |  |  |  |  |  |
| Non-Alcoholic Fatty Liver Disease | 0.000 | 0.000 | Inf | 0.997 |  |  |  |  |  |
| Stage | 2.610 | 1.541 | 4.421 | 0.000 |  | 1.909 | 1.090 | 3.342 | 0.024 |
| Grade | 1.390 | 0.812 | 2.381 | 0.230 |  |  |  |  |  |
| GLncM | 0.456 | 0.256 | 0.815 | 0.008 |  | 0.544 | 0.300 | 0.987 | 0.045 |

HR: hazard ratio; OS: overall survival.
