# Supplementary figures and images for: Development and validation of a genomic instability-related lncRNA prognostic model for hepatocellular carcinoma
Source: Front Genet. 2023 Jan 12;13:1034979. doi: 10.3389/fgene.2022.1034979 (PMC9877230; doi:10.3389/fgene.2022.1034979)

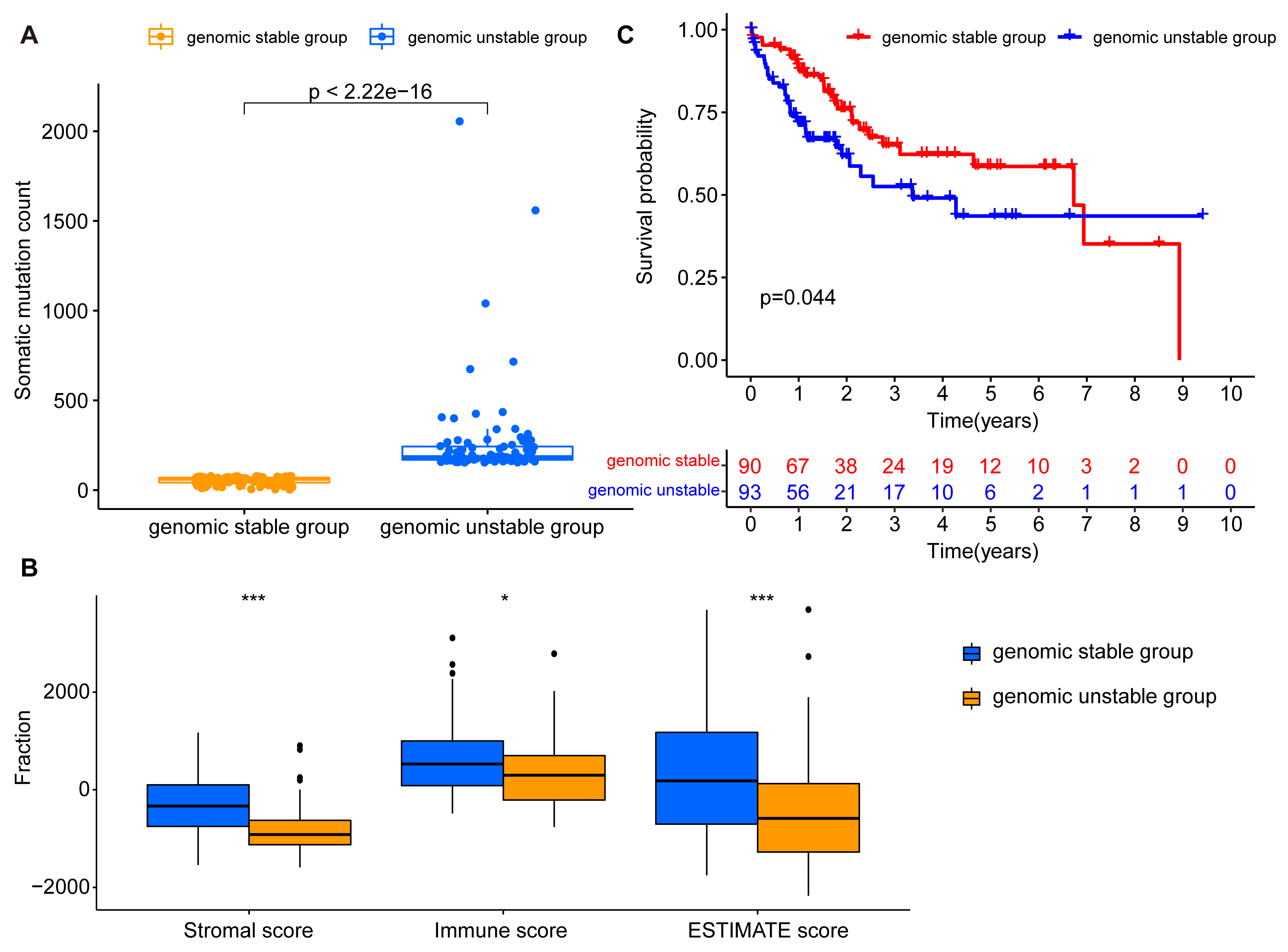

Supplement: Supplementary file 2 [file Image3.tif]

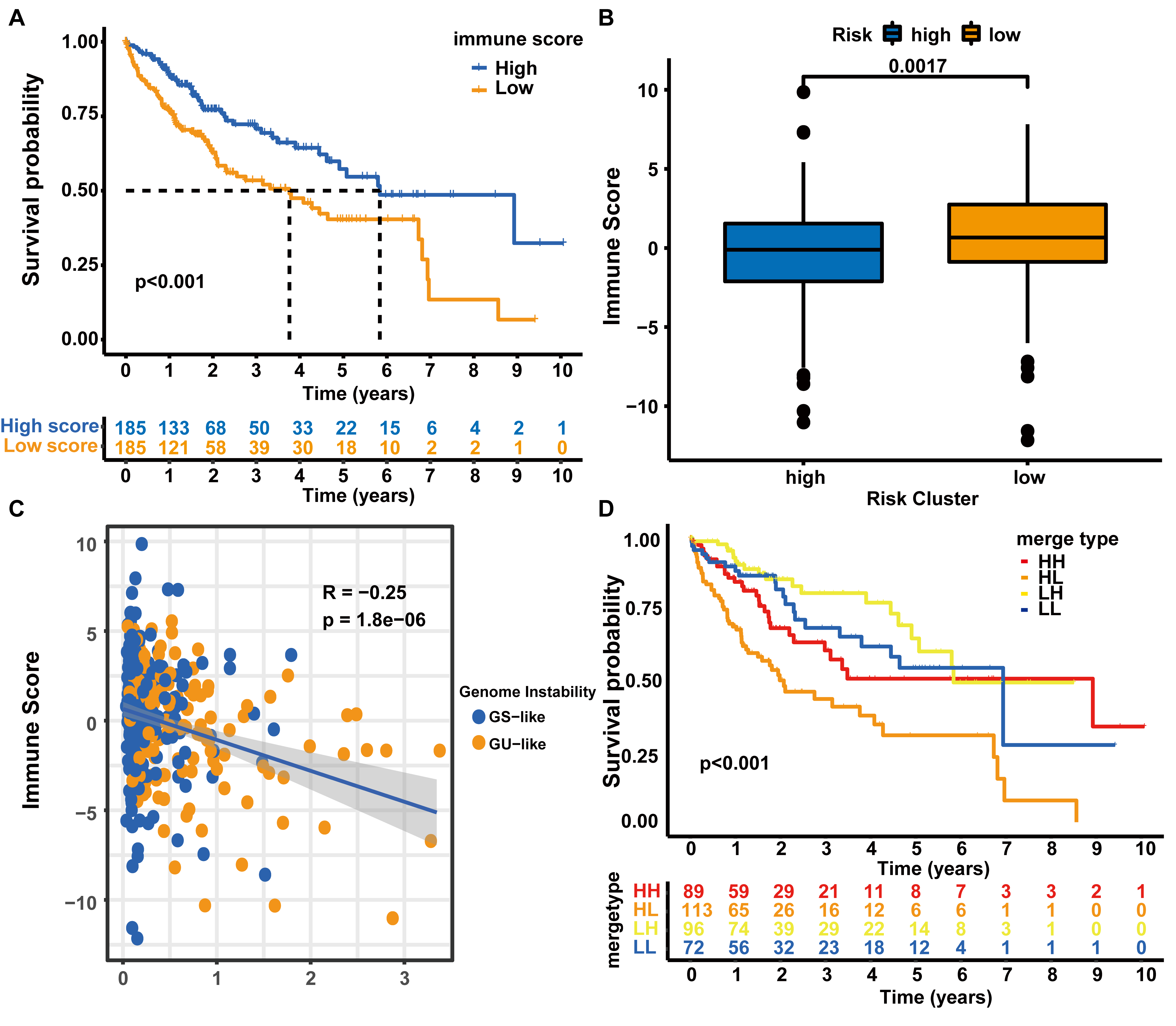

Supplement: Supplementary file 3 [file Image2.tif]

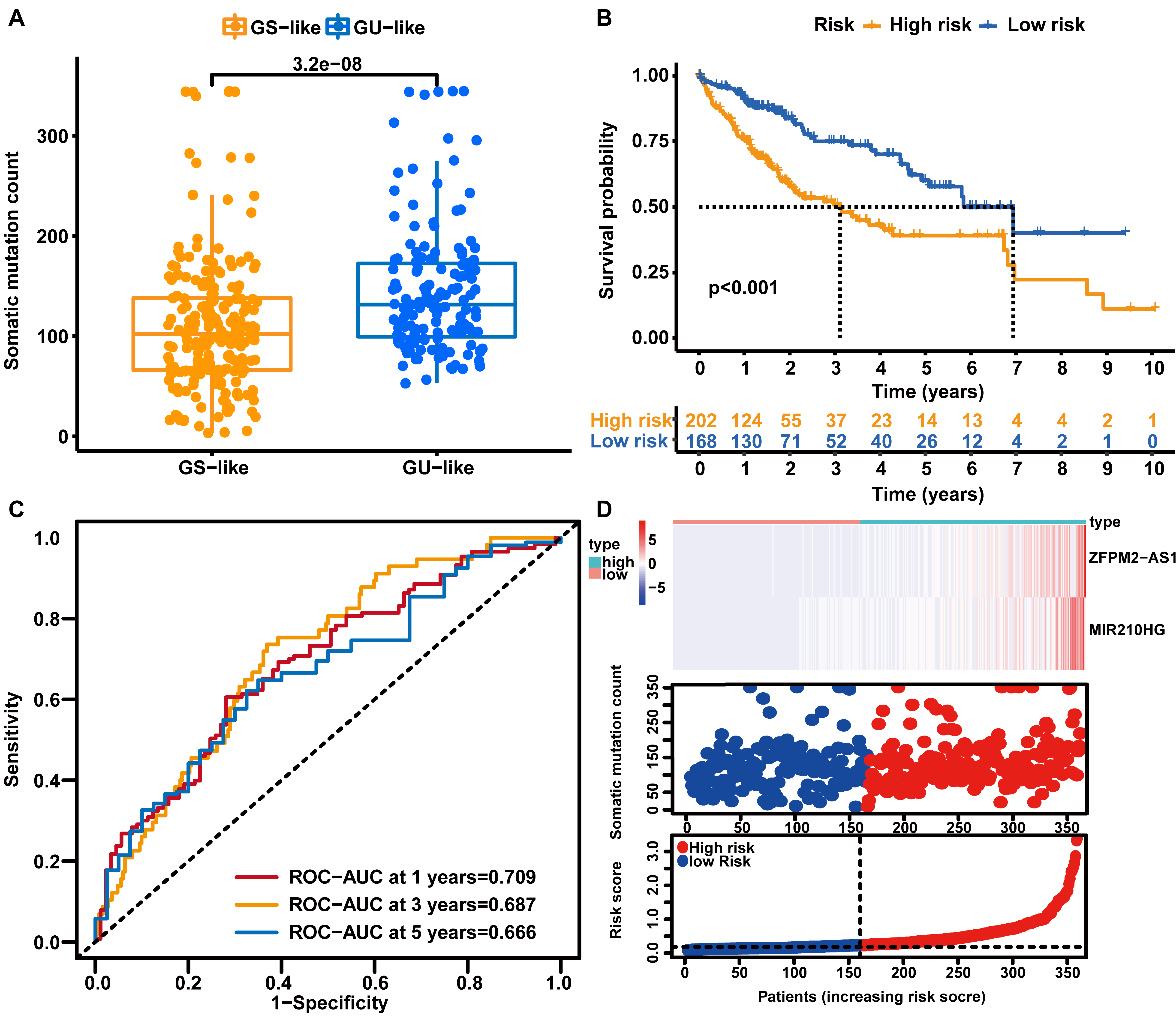

Supplement: Supplementary file 4 [file Image1.tif]
